# A pilot study to assess the feasibility of evaluation of markers of response to chemotherapy at one day & 21 days after first cycle of chemotherapy in carcinoma of breast: a prospective non-randomized observational study

**DOI:** 10.1186/1477-7819-7-35

**Published:** 2009-03-30

**Authors:** Shekhar Sharma, KR Hiran, K Pavithran, DK Vijaykumar

**Affiliations:** 1 Department of Surgical Oncology, Amrita Institute of Medical Sciences & Research Center, Amrita Lane, Edapally, Ernakulam – 682026, Kerala, India; 2 Department of Pathology, Amrita Institute of Medical Sciences & Research Center, Amrita Lane, Edapally, Ernakulam – 682026, Kerala, India; 3 Department of Medical Oncology, Amrita Institute of Medical Sciences & Research Center, Amrita Lane, Edapally, Ernakulam – 682026, Kerala, India

## Abstract

**Background:**

Interest in translational studies aimed at investigating biologic markers in predicting response to primary chemotherapy (PCT) in breast cancer has progressively increased. We conducted a pilot study to evaluate feasibility of evaluating biomarkers of response to PCT at one & 21 days after first cycle.

**Methods:**

Adult, non-pregnant, non-lactating women with histologically confirmed infiltrating duct carcinoma underwent serial core biopsies after first cycle of PCT and these were scored for *Ki*-67, *Bcl*-2 and *Caspase*-3 using immunohistochemistry.

**Results:**

We recruited 30 patients with a mean age of 51 years. We were successful 95.6% times in performing a core biopsy and of these 84.6% had adequate tissue in the cores harvested. After a mean of 4 cycles of PCT, 26 patients underwent surgery and good response was noted in 9 patients (30%) using Miller-Payne criteria. There was a trend noted in all markers, which appeared different in those with good response and poor response. Good responders had significantly higher *Ki-67 *and significantly lower *Bcl*-2 at baseline and a significant decrease in *Ki-67 *and *Caspase-3 *at 21 days after the first chemotherapy.

**Conclusion:**

We report a detectable change in biomarkers as early as 24–48 hours after the first chemotherapy along with a definite trend in change that can possibly be used to predict response to chemotherapy in an individual patient. The statistical significance and clinical utility of such changes needs to be evaluated and confirmed in larger trials.

## Background

There is increasing interest in the ways and means to predict the response of an individual patient to primary chemotherapy (PCT) with an ultimate interest to predict individual responses to treatment in the minimum time feasible.

Clinical response has been used as an intermediate, surrogate end-point for assessment of the efficacy of PCT in an individual, although this assessment is far from accurate [1]. Tools are, therefore, required to better assess the efficacy of chemotherapy regimen.

Ellis et al showed that chemotherapy induced apoptosis in early breast cancer could be demonstrated soon after the chemotherapy [2]. In continuation of this, it would indeed be useful to have a marker of response that can be evaluated as soon as possible after the first cycle of chemotherapy and correlates to the clinical outcome.

We wanted to evaluate if it was feasible to harvest a satisfactory core biopsy immediately after first cycle and just prior to second cycle of chemotherapy, when patient is available in the hospital along with feasibility to evaluate biomarkers of response to chemotherapy in these biopsies. The correlation to response, if proven, would help clinicians to tailor chemotherapy to individual patients and may provide the opportunity to offer earlier possible alternative, non-cross-resistant regimens to those patients not achieving a response to the initial regimen.

We proposed to explore the change in biomarkers of response to PCT at one day and 21 days after the first cycle of PCT in women with breast cancer attending our institution for care and towards this aim we initiated a pilot study in our institution, after appropriate scientific and ethics committee approval, in the patients of breast cancer undergoing PCT. The primary aims of this pilot study were to assess the feasibility and reproducibility of performing:

a) Serial core biopsies one day and 21 days after first cycle of chemotherapy, with emphasis on patient acceptance and complications of the procedure.

b) Assays of apoptosis (*Caspase-3 *&*Bcl-2*) and proliferation index (*Ki-67*) in patients of carcinoma of breast on core biopsy specimens using immunohistochemistry (IHC).

c) Quantification of extent of change in these biomarkers of response to chemotherapy one day and 21 days after first cycle of chemotherapy.

d) Histopathological response grading at final surgical histopathology using Miller-Payne response assessment criteria.

## Patients and methods

Adult (more than 18 years of age) non-pregnant, non-lactating women with histologically confirmed, previously untreated infiltrating duct carcinoma (IDC) of breast who were advised PCT, as per institutional protocol, were eligible. Patients with inflammatory breast cancer or those with history of any indigenous form of therapy for breast cancer were excluded from this study. The study was approved by the Institute review board.

After an informed written consent, serial core biopsies were taken before (C0 biopsy), 24–48 hours (C1 biopsy) and 21 days (C2 biopsy) after first cycle of chemotherapy. Chemotherapy regimen was at discretion of the treating medical oncologist. Serial core biopsies were obtained exclusively for the purpose of this study for determination of potential predictive surrogate markers of response. A core biopsy was obtained using Bard Monopty disposable biopsy instrument (Covington, GA). Three core biopsies were taken – first before starting chemotherapy (C0), second 24–48 h after cycle one (C1), and third 21 days after cycle one (C2). Biopsy specimens, two cores each time, were fixed in 10% buffered formalin and embedded in paraffin and sectioned into 4 μm-thick sections.

Surgery was scheduled after completion of 2–6 cycles of PCT according to patient's response to chemotherapy and at discretion of the treating physicians. The study pathologist carefully evaluated the definitive surgical specimen for the presence of residual disease and grading of pathological response to chemotherapy was done using Miller-Payne criteria for assessment of response to chemotherapy [3]. Miller-Payne response grade 4 & 5 were considered as good pathological response (GPR) while grades 1 to 3 were considered poor pathological response (PPR). *ER, PR*, and *HER2/neu *were evaluated only on C0 biopsy.

Markers for proliferation (*Ki-67*), *Caspase-3 *and *Bcl-2 *were evaluated by IHC using appropriate antibodies (Table 1). Slides were deparaffinized and hydrated. Standard techniques for antigen retrieval, blocking endogenous peroxidase activity and nonspecific antibody binding were followed before immuno-staining with commercially available antibodies (Table 1). Primary antibodies were pre-diluted except for *Caspase-3 *for which a dilution of 1:500 was used. Incubation period for all the antibodies were 1 hour except *Ki-67 *that was kept for 2 hours at 37°C. Known positive and negative controls were included for each batch run. Slides were scored for percentage of positive cells and relative intensity.

**Table 1:** Details of IHC antibodies for *Caspase-3, Bcl-2 *and *Ki-67*

**Antigen**	**Antibody**	**Manufacturer**	**Scoring**
*Ki-67*	MAb	Zymed, San Francisco, CA	Nuclear staining; % positive

*Bcl-2*	MAb	DAKO, Carpenteria, CA	Cytoplasmic staining, % positive

*Caspase-3*	Mouse	Imgenex, San diego, CA	Nuclear and cytoplasm staining, % positive

MAb – Monoclonal antibody

The feasibility of performing serial core biopsies was not addressed statistically. Non-parametric tests were applied to assess the other variables. Patient baseline characteristics, the treatment regimen, and molecular markers were each assessed for an association with pathologic response using the Mann-Whitney U test. The change in biomarkers of response from pre-treatment was assessed in the GPR & PPR groups by paired comparisons, using the Wilcoxon signed rank test, while within group analysis was performed using Wilcoxon rank sum test.

## Results

We recruited 30 patients of breast cancer with a mean age of 51 years ( ± 8.4) for this study from April 2007 to June 2008.

Patient demographics are mentioned in Table 2. Disease characteristics are mentioned in Table 3. There were no significant differences in demographic pattern between GPR & PPR groups.

**Table 2:** Patient demographics

**Characteristic**	**Mean ( ± SD)**	**Range**
Age (years)	51 ( ± 8.4)	31–63

Duration of symptoms (months)	10.54 ( ± 10.88)	0.25 – 40

Age at Menarche (years)	14.16 ( ± 1.7)	11–18

Age at Marriage (years)	21.58 ( ± 4.26)	13–33

Age at menopause (years)	47.58 ( ± 3.73)	41–54

Parity (median)	2	0–5

Age at first childbirth	23.81 ( ± 4)	17–32

Duration of breast feeding (months)	43.22 ( ± 22.02)	6–96

Number of PCT cycles (median)	4	3–9

Menstrual status		

*Premenopausal*	19	63.3

*Postmenopausal*	11	36.7

Laterality		

*Right*	18	60

*Left*	11	36.7

*Bilateral*	1	3.3

**Table 3:** Disease characteristics

**Parameter**		**N**	**Percent**
Stage of disease at presentation			

*II B*		4	13.3

*III A*		7	23.3

*III B*		5	16.7

*III C*		4	13.3

*IV*		10	33.3

Histological type			

*Infiltrating ductal carcinoma (IDC)*		27	90.0

*Infiltrating lobular carcinoma (ILC)*		2	6.7

*Combined ILC & IDC*		1	3.3

Grade of tumor			

*Low grade*		4	13.3

*Intermediate grade*		14	46.7

*High grade*		12	40.0

Miller-Payne response ^#^			

Poor	Grade I (no or <10% response)	5	16.7
	
pathological	Grade II (10–30% response)	4	13.3
	
response (PPR)	Grade III (30–90% response)	8	26.6

Good pathological	Grade IV (>90% response)	4	13.3
	
response (GPR)	Grade V (complete response or few isolated tumor cell islands remaining)	5	16.7

#: Miller Payne response could not be assessed in 4 patients who did not undergo surgery

ER, PR and Her-2/neu receptors were all positive in four (13.3%) patients while all three were negative in 10 (33.3%) patients. Of the 10 patients (33.3%) with Stage IV disease, whole body skeletal scintigraphy detected metastasis in eight patients (26.7%); chest X-ray in one patient (3.3%); ultrasound abdomen in four patients (13.3%) and CT scan chest in two patients (6.7%).

We were successful in harvesting core biopsy tissue with adequate cellularity in a reasonable proportion of patients (Table 4). Of the proposed 90 core biopsy procedures (three each in 30 patients), only four patients (4.44%) refused the third core biopsy (C2) due to procedure related pain. We did not observe any other procedure related complications.

**Table 4:** Feasibility & adequacy of core biopsy procedures

**Procedure**	**N**	**%**
Core biopsy at baseline (C0 biopsy)	30	100

Core biopsy 24–48 hours after first cycle of chemotherapy (C1 biopsy)	30	100

Core biopsy 21 days after first cycle of chemotherapy (C2 biopsy)	26	86.6

Adequacy		

At C0 biopsy	30	100

At C1 biopsy	28	93.3

At C2 biopsy	22	73.3 (84.3% of 26 attempted)

4 (13.3%) refused C2 biopsy; 4 (13.3%) had paucicellular harvest on C2 biopsy		

**Definitive surgery**	26	86.6

4 (13.3%) patients did not undergo surgery due to different reasons.		

Although paucicellular harvest can be attributed to poor technique and less number of cores taken, it is interesting to note that out of the four patients who had a paucicellular harvest at 21 days after chemotherapy (C2 biopsy), three had a good response on final histopathology by Miller-Payne criteria.

Chemotherapy regimens included Adriamycin & Cyclophosphamide followed by Paclitaxel (AC+T) in 12 patients (40%); combination of Docetaxel, Adriamycin and Cyclophosphamide (TAC) in 10 patients (33.3%); 5-Flurouracil, Adriamycin (or Epirubicin) and Cyclophosphamide (FAC/FEC) in 6 patients (20%) and Docetaxel alone in 2 patients (6.7%)

Miller-Payne pathological response category could not be assessed in 4 patients (13.3%) (one expired, one had progressive disease on chemotherapy, one refused surgery and one had surgery cancelled due to chemotherapy induced cardiomyopathy). Details of Miller-Payne pathological response in the remaining 26 patients are shown in Table 3. Nine patients (30%) had a GPR to chemotherapy (Table 3).

**Figure 1:**
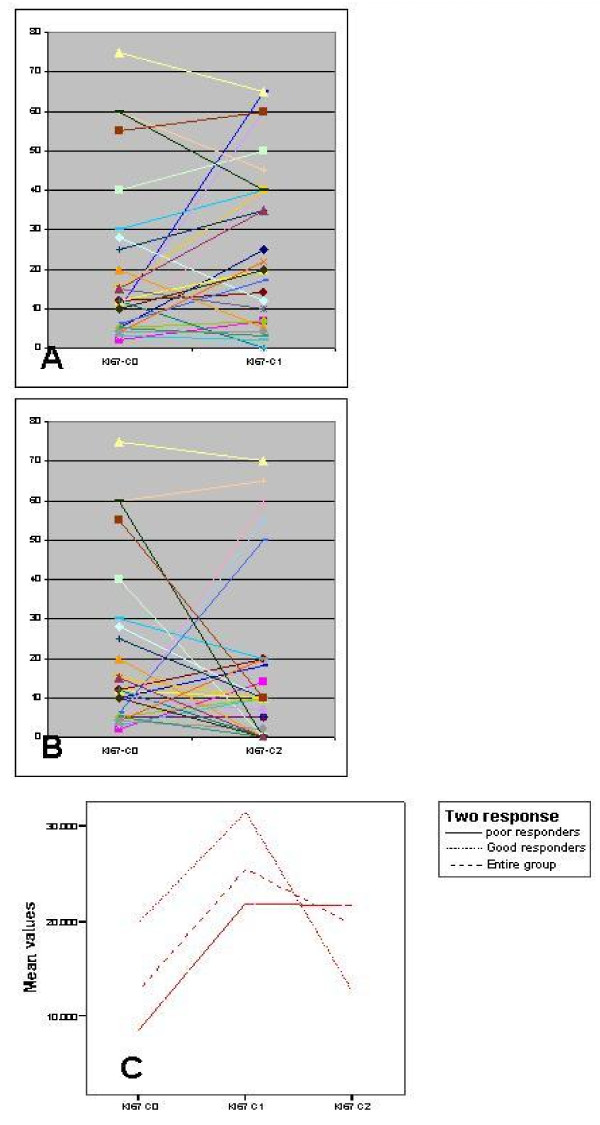
**Comparison of Ki-67 levels in (a) C0–C1 biopsy; and (b) C0–C2 biopsy; and (c) Change in mean value over time**.

**Figure 2:**
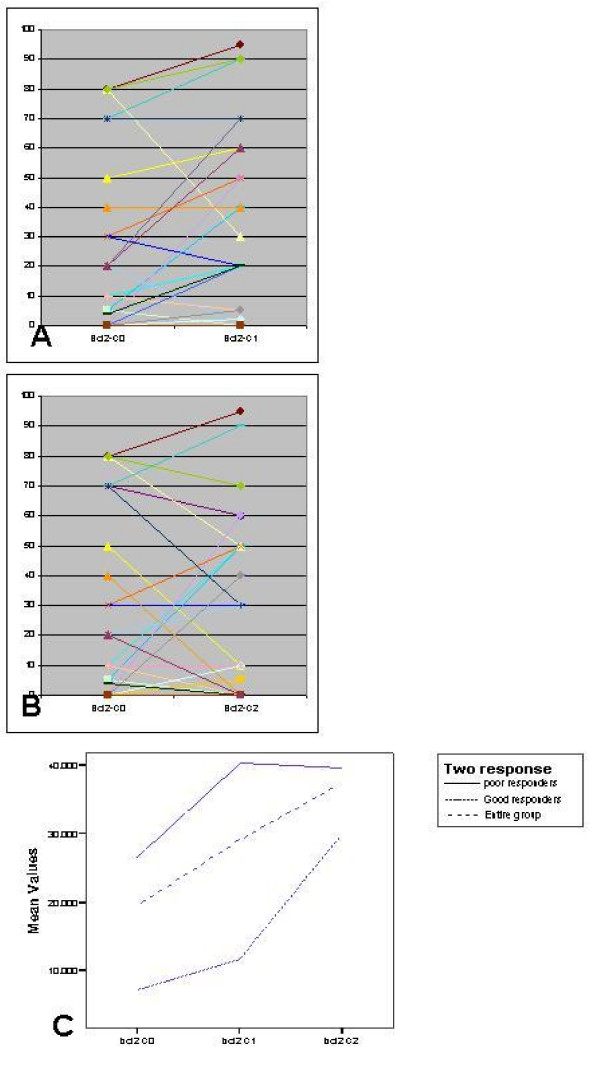
**Comparison of Bcl-2 levels in (a) C0–C1 biopsy; and (b) C0–C2 biopsy; and (c) Change in mean value over time**.

**Figure 3:**
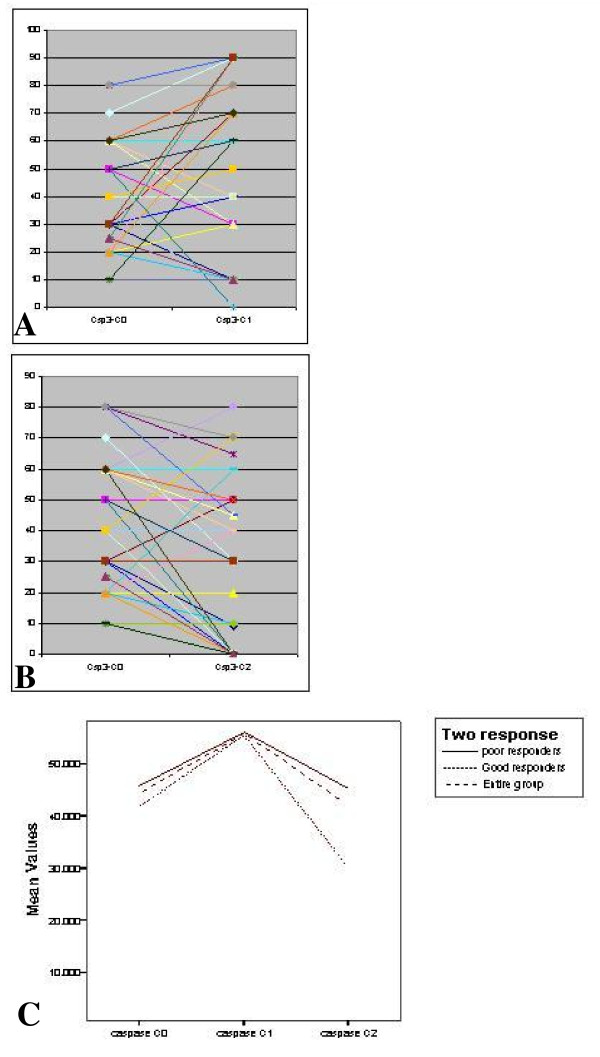
**Comparison of Caspase-3 (Csp-3) levels in (a) C0–C1 biopsy; and (b) C0–C2 biopsy; and (c) Change in mean value over time**.

Levels of *Ki-67, Bcl-2*, and *Caspase*-3 and their comparisons in C0, C1 and C2 biopsy are shown in Figures 1, 2 and 3 respectively.

We observed that GPR group had significantly higher *Ki-67 *at baseline (p = 0.042) and both GPR & PPR groups showed a rise at 24–48 hours after first chemotherapy (in C1 biopsy). This decreased 21 days after first chemotherapy to below the baseline values (in GPR group) as well as C2 values in PPR group, which were static at C1 levels. The difference between GPR & PPR groups in levels of *Ki-67 *seen in C2 biopsy was not significant (p = ns), although the difference in change from C1 to C2 appears striking with a steep slope in GPR group (Figure 1c).

On the converse, *Bcl-2 *was significantly lower in GPR group in all the three biopsies (p = 0.015; 0.014; 0.039 for C0, C1 & C2 respectively). Chemotherapy induced a steady rise in the entire group, which was steeper in GPR group from C1 to C2. *Bcl*-2 peaked at biopsy taken at 24–48 hours after the first cycle in the PPR group and then had a plateau to nearly same level at 21 days (Figure 2c).

*Caspase-3 *values peaked at 24–48 hours before falling to near baseline levels at 21 days after the first chemotherapy with nearly similar baseline and peak values in both the groups (p = ns for both C0 & C1 biopsies). The decline in GPR group for values of *Caspase*-3 from C1 to C2 biopsy was steeper, the difference from PPR group being significant (p = 0.024) at this point. In the GPR group, the *Caspase-3 *values at C2 fell below the baseline values (C0) (Figure 3c).

In our study, the magnitude of change in *Bcl-2 *within 24–48 hours after the first chemotherapy, in the entire group, was significant (p = 0.04), while that for markers of proliferation (*Ki-67*) and apoptosis (*Caspase-3*) was not significant (p = ns).

Thus, tumors with a higher *Ki-67 *at baseline along with a low *Bcl-2 *(anti-apoptotic gene) responded better to chemotherapy. In other words, high rates of apoptosis and proliferation at baseline were associated with improved pathological response. Another interesting observation during this study was that at 21 days, a decrease in *Ki-67 *and *Caspase-3 *was predictive of favorable response (p = 0.01 for both).

In this study, *ER*-positive tumors had a significant association with poor response (p = 0.014) and had a higher *Bcl-2 *expression at baseline (mean *Bcl-2 *35.38 in *ER*-positive vs. mean *Bcl-2 *14.35 in ER negative tumors; p = 0.04). There was no difference in expression of *Ki-67 *or *Caspase-3 *in *ER*-positive or negative tumors or in expression of these markers or in response between *PR *and *Her2/neu *positive or negative tumors.

As a word of caution, p values of significance should be interpreted with caution due to the small sample size. It was primarily aimed as a pilot study to verify feasibility and reproducibility of this trial design and to see if changes in biomarkers could be measured and quantified at patient-friendly time points, aims that it apparently has achieved.

We faced problems using *Caspase-3 *to evaluate the apoptotic index, as this terminal enzyme of the apoptotic cascade is cytoplasmic in location. This led to a diffuse staining of slides, which caused difficulty in interpretation of positive cells and percentage positivity. Additionally, technical expertise in slide preparation and IHC staining were other major hurdles in the initial phase of the study.

On the basis of this pilot study, we observe that this trial design is feasible (in this context, patient acceptable without any specific objective incentive) and quantification of biomarkers of response to chemotherapy can be performed on these core biopsies. There is a trend towards change noted in these markers (in this study, *Ki*-67, *Bcl*-2, *Caspase*-3) both at 24 hours and at 21 days after the first cycle of chemotherapy, although these results need to be confirmed in larger studies. In our experience, Miller-Payne criteria to assess response to chemotherapy, is an easily reproducible method of grading response objectively.

We hope that we will be able to improve the adequacy of tissue by increasing the number of cores harvested each time from two in the present study to three or four in future studies. A more proactive approach to pain medication prescription will, hopefully, help us in preventing dropouts in further trials. However, we would need alternative methods of evaluating apoptotic index (eg TUNEL, etc) due to problems associated with *Caspase-3 *in any future study.

Whether we need to repeat the same design (two biopsies after baseline – one 24–48 hours and second 21 days after first chemotherapy) or either one of these biopsies can be omitted is a matter of debate, although in our opinion a three point measurement will improve the predictive power of the larger trial.

## Discussion

Pathological complete response to PCT has been correlated with long-term outcome [4,5], although this is seen in only 3–30% of patients [6]. Bio-molecular predictors of tumor response to primary CT include S-phase fraction, ER, PgR, thymidine labeling index, ploidy, p53 and c-erbB-2 (Her-2/neu) [7-12].

There is preliminary evidence that supports proliferation & apoptosis-related markers as predictors of long-term response to PCT [13,14]. These include, among others, markers for induction of apoptosis, expression of *Bcl-2*, and proliferation index (*Ki-67 *assay) [2,15,16]. However the exact relationship of the levels of biomarkers in a tumor in pre and post chemotherapy setting is relatively under-explored.

Studies have usually evaluated markers for response to chemotherapy after a significant delay [2]. A time gap of 10 days or more poses a difficult hurdle for investigators to have the patient come back again for tissue harvesting alone with most patients being reluctant to do so in absence of any objective incentive for their extra time, effort and expenses. This is a more acute issue in the Indian perspective, where patients often need to travel great distances to seek medical care.

We chose to evaluate three biomarkers, namely *Ki-67 *(marker of proliferation), *Bcl-2 *and *Caspase-3 *(anti- and pro-apoptotic markers) as data exist showing a close relationship between apoptosis and proliferation in untreated tumours [17,18]. The decision to restrict the number of biomarkers to three was to keep the study design as simple as possible in the pilot trial.

Several groups have found that *Ki-67 *decreases after chemotherapy over a variable duration [19]. Some studies have demonstrated a relationship of change in *Ki-67 *with response [15,20]. In a similar pilot study where *Ki-67 *was measured in 20 patients treated with chemo-endocrine therapy (mitoxantrone, mitomycin C, methotrexate and tamoxifen), a decrease at day 10 or 21 after the first course of treatment correlated with response at 3 months (*p *= 0.008). *Ki-67 *changes between the responders and non-responders were significant for both absolute and percentage change in the chemotherapy (*p *= 0.01 and *p *= 0.005, respectively) as well as in chemo-endocrine therapy group (*p *= 0.03 and *p *= 0.06, respectively) [21]. Further follow up showed that this decrease in *Ki-67 *after 10–21 days of therapy had a significant association with good clinical response on univariate analysis [15]. While significant associations with response have been revealed in these studies, none have assessed the predictive power in individual patients.

Whilst some studies have shown that a high proliferative index is a poor prognostic indicator [22,23], others have debated this with observations that patients with highly proliferative tumours respond well to chemotherapy [24]. Honkoop et al showed that a high proliferative index in residual tumours after neoadjuvant chemotherapy and endocrine therapy was associated with a decreased disease free survival [25].

In this study, we noted that a higher baseline *Ki-67 *was associated with better response to chemotherapy, probably because a higher fraction of these proliferative tumors at initiation of chemotherapy were susceptible to chemotoxic effects. The low 21-day *Ki-67 *values, in good responders, similar to those reported in literature, are indirect evidence of the efficacy of the chemotherapy in these patients in eliminating the mitotic fraction. It is intriguing to note that, as soon as 24 hours after chemotherapy, there was a rise in *Ki-67 *levels, something that, to our knowledge, has not been reported in literature.

*Bcl-2 *gene encodes for a 26-kDa protein that mainly inhibits apoptosis. However, the role of *Bcl-2 *expression on clinical outcome following chemotherapy is still under investigation, since available data are in some instances contrasting [26]. Also, interpretation of treatment benefit as a function of biomarkers is difficult in the absence of randomized, controlled trials.

A number of studies, covering about 5000 patients, with breast cancer at different stages showed that *Bcl-2 *over-expression correlated to a differentiated phenotype and a favorable prognosis in patients subjected to local-regional, hormonal or cytotoxic therapies [14,27].

Our data suggests that breast carcinomas with low baseline apoptosis may respond poorly to chemotherapy. We observed a significant inverse correlation between expression of *Bcl-2 *and response to the chemotherapy. These results are in general line with the postulated anti-apoptotic function of *Bcl-2 *gene, higher levels in poor responders indicating a possible immunity from chemotherapy induced apoptosis.

Some possible explanations for these paradoxical results have been mentioned in literature and include a complex interaction of p53 or its mutant variations with *Bcl-2*, an inhibitory effect of *Bcl-2 *on proliferation along with regulation of *Bcl-2 *expression by estrogen and presence of antagonists, which may negate its anti-apoptotic function [13,28].

The prognostic and predictive value of apoptotic markers in breast cancer is not yet fully understood. There is some suggestion that apoptotic index is an independent prognostic factor. Our results are similar to other reports in the literature that chemotherapy induces early changes in apoptosis [2].

Data from this study and another similar study [29] suggest that it may be possible, in future, to determine, as early as 24–48 h after administration of chemotherapy, whether a woman is likely to respond to a specific agent or not, information that might help to make an early decision regarding any change in such treatment. The novel approach in this study can also answer questions regarding the role of other markers and response to individual therapies.

This study does have a few limitations like small sample size (30 patients were recruited as this was planned as a pilot study only), heterogeneous patient population (no stratification on the basis of receptor status, chemotherapeutic regimen received or stage of disease) all of which in themselves can argue for a different disease biology and consequently difference in responses to chemotherapy. However, even with these limitations, results are impressive enough to favor larger, more rigorously controlled trials to confirm these.

## Conclusion

In summary, we present a clinical design incorporating sequential core biopsy after first cycle of PCT in breast cancer that can be used as a model in future trials to correlate surrogate end point biomarkers with response. The model can also be used to incorporate novel agents with standard treatments. Changes in biomarkers like apoptosis and proliferation can then, if validated with larger trials using standard regimens, be used to determine the efficacy and/or superiority of the novel combinations compared to standard treatments.

Whether or not trends observed in this study are significant and whether these can be used to tailor chemotherapy (our ultimate aim) awaits larger trials. Further studies, including a larger sample size receiving single standardized chemotherapy regimen, are warranted, especially in a prospective manner with uniform methods of measurement and cut-off points to assess the potential value of molecular markers in clinical practice. These studies will need to include multiple assays such as nuclear grade, levels of expression of p53, markers for cell proliferation, multi-drug resistance, and apoptosis [30].

## Competing interests

The authors declare that they have no competing interests.

## Authors' contributions

SS was instrumental in design the concept, patient recruitment, data analysis, manuscript preparation and editing. HKR was instrumental in designing the trial, evaluation of slides for data generation, manuscript preparation and editing. PK, and DKV were instrumental in ratifying study design, patient recruitment, literature search and manuscript editing & final approval. All authors accept the responsibility of contents of this manuscript.
